# Quantitation of mitral regurgitation using positron emission tomography

**DOI:** 10.1186/s13550-024-01150-1

**Published:** 2024-09-18

**Authors:** Jonathan Sigfridsson, Tomasz Baron, Johannes Bergsten, Hendrik J. Harms, Jonny Nordström, Tanja Kero, Patrik Svanström, Elin Lindström, Lieuwe Appel, My Jonasson, Mark Lubberink, Frank A. Flachskampf, Jens Sörensen

**Affiliations:** 1 Molecular Imaging and Medical Physics, Department of Surgical Sciences, Uppsala University, Uppsala, Sweden; 2 Cardiology and Clinical Physiology, Department of Medical Sciences, Uppsala University, Uppsala University Hospital, Uppsala, Sweden; 3 Uppsala Clinical Research Centre, Uppsala University, Uppsala, Sweden; 4 MedTrace Pharma A/S, Horsholm, Denmark; 5 Centre for Research and Development, Uppsala/Gävleborg County, Gävle, Sweden

**Keywords:** Cardiovascular diseases, Mitral regurgitation, Cardiac PET, Cardiovascular MRI, Quantitation

## Abstract

**Background:**

Cardiac positron emission tomography (PET) offers non-invasive assessment of perfusion and left ventricular (LV) function from a single dynamic scan. However, no prior assessment of mitral regurgitation severity by PET has been presented. Application of indicator dilution techniques and gated image analyses to PET data enables calculation of forward stroke volume and total LV stroke volume. We aimed to evaluate a combination of these methods for measurement of regurgitant volume (RegVol) and fraction (RegF) using dynamic ^15^O-water and ^11^C-acetate PET in comparison to cardiovascular magnetic resonance (CMR).

**Results:**

Twenty-one patients with severe primary mitral valve regurgitation underwent same-day dynamic PET examinations (^15^O-water and ^11^C-acetate) and CMR. PET data were reconstructed into dynamic series with short time frames during the first pass, gated ^15^O-water blood pool images, and gated ^11^C-acetate myocardial uptake images. PET-based RegVol and RegF correlated strongly with CMR (RegVol: ^15^O-water *r* = 0.94, ^11^C-acetate *r* = 0.91 and RegF: ^15^O-water *r* = 0.88, ^11^C-acetate *r* = 0.84, *p* < 0.001). A systematic underestimation (bias) was found for PET (RegVol: ^15^O-water − 11 ± 13 mL, *p* = 0.002, ^11^C-acetate − 28 ± 16 mL, *p* < 0.001 and RegF: ^15^O-water − 4 ± 6%, *p* = 0.01, ^11^C-acetate − 10 ± 7%, *p* < 0.001). PET measurements in patients were compared to healthy volunteers (*n* = 18). Mean RegVol and RegF was significantly lower in healthy volunteers compared to patients for both tracers. The accuracy of diagnosing moderately elevated regurgitant volume (> 30mL) was 95% for ^15^O-water and 92% for ^11^C-acetate.

**Conclusions:**

LV regurgitation severity quantified using cardiac PET correlated with CMR and showed high accuracy for discriminating patients from healthy volunteers.

**Supplementary Information:**

The online version contains supplementary material available at 10.1186/s13550-024-01150-1.

## Introduction

Primary mitral valve regurgitation is a common valve disease with increasing prevalence, primarily affecting the elderly [1–3]. Mitral regurgitation leads to left ventricular (LV) remodeling and eventually dysfunction. The treatment options are surgical valve repair, replacement or percutaneous intervention. Correct timing of treatment is a complex task that requires monitoring with cardiac imaging. In routine practice, echocardiography is the preferred method, and image findings are combined with clinical assessment [4, 5]. For evaluating the severity of mitral regurgitation, several qualitative signs as well as measurement of regurgitant volume (RegVol) and regurgitant fraction (RegF) are performed. LV volumes, and LV ejection fraction (LVEF) are recommended for guiding decisions. The gold standard for these assessments is cardiovascular magnetic resonance (CMR) [6].

Recent evidence suggests that besides the standard measurements, additional assessment of myocardial performance is important [7]. Signs of myocardial damage and remodeling is likely an earlier indication of decompensated valvular disease [8–10]. In light of this, despite of its limited use today, molecular imaging with positron emission tomography (PET) could be of increased relevance in mitral regurgitation. In a recent study, our group evaluated myocardial external efficiency as an outcome variable in severe mitral regurgitation using ^11^C-acetate PET [11]. PET added incremental information to CMR measurements of regurgitant magnitude, and was a significant predictor of outcome on its own. A dual-modality approach of CMR and ^11^C-acetate PET would be a powerful tool, but resource demanding. It is therefore of interest to investigate the use of stand-alone PET to assess regurgitation severity.

Calculation of the parameters needed for such evaluations has previously been studied using dynamic ^11^C-acetate imaging. First-pass analysis and indicator dilution techniques enable quantification of cardiac output (CO) and forward stroke volume (FSV) [12, 13]. Electrocardiography (ECG)-gating of ^11^C-acetate late uptake images can provide LV total stroke volume (LVSV) from the same acquisition [14]. Hence, using a combination of indicator dilution techniques and ECG-gating, it should be possible to estimate RegVol and RegF from one dynamic ^11^C-acetate PET scan.

As cardiac PET is mainly utilized to evaluate ischemic heart disease, it would likely be further beneficial to use clinically important perfusion tracers for this purpose. ^15^O-water is considered the reference method for quantifying myocardial blood flow (MBF) [15–17]. CO and FSV can be calculated from dynamic ^15^O-water PET using the same methods as presented for ^11^C-acetate [13]. ECG-gating of ^15^O-water for assessment of LVSV constitutes a challenge since water is freely diffusible and yields no retention images. However, methods for blood pool-based gating are feasible [18, 19]. As such, RegVol and RegF measurements should also be attainable with ^15^O-water PET.

In this study, we aimed to investigate using both a retention PET tracer (^11^C-acetate) and a freely diffusible PET tracer (^15^O-water), for quantitation of RegVol and RegF, as compared to CMR.

## Methods

### Study design and population

The patient data used in the current study have been described in detail previously [11]. We utilized a sub-cohort of this previous study, including only subjects where ECG-gated ^15^O-water data were available. All patients were diagnosed with asymptomatic severe degenerative and chronic mitral regurgitation, as according to echocardiographic criteria. Subjects with more than mild concomitant valve diseases were excluded. Patients underwent ^15^O-water PET, ^11^C-acetate PET and CMR examinations on the same day. PET and CMR scans were conducted within one hour, avoiding fluid intake between scans to minimize changes in hemodynamic loading conditions.

In addition, 21 healthy volunteers were included and underwent ^15^O-water PET. Five of these additionally underwent ^11^C-acetate PET and echocardiography on the same day. Healthy volunteers had no history of cardiovascular disease or symptoms at the time of the examinations.

Imaging data were acquired in three studies, all approved by the Regional Ethical Review Board at Uppsala University (Dnr: 2012 − 543, 2020–07017, 2021–05230). The examinations were performed at Uppsala University Hospital between 2016 and 2023 and all subjects provided written informed consent.

### PET scanning procedure

PET examinations were conducted with a digital PET/CT scanner (Discovery MI, GE Healthcare) with an axial detector coverage of 20 cm (DMI-20: all patients, 5 healthy volunteers with ^15^O-water and ^11^C-acetate scans) or 25 cm (DMI-25: 16 healthy volunteers with ^15^O-water scans). The scans were performed in the resting state and a 4-lead ECG was connected during the procedure. Manual heart rate recording was performed before the start of the scans and during first pass.

A low dose CT was acquired for attenuation correction and anatomical localization. Subsequently, 400 MBq of ^15^O-water was injected in an antecubital vein using an automated bolus (10 mL at 0.8 mL/s followed by 30 mL saline at 2.0 mL/s) simultaneous with the start of a 4 min PET-acquisition in list-mode. After approximately 15 min post the ^15^O-water injection, 5 MBq/kg ^11^C-acetate was administered using the same injection protocol and a 27 min list-mode acquisition.

List-mode PET data were reconstructed into dynamic series (^15^O-water: 1 × 10, 8 × 5, 4 × 10, 2 × 15, 3 × 20, 2 × 30s and ^11^C-acetate: 12 × 5, 6 × 10, 4 × 30, 4 × 60, 2 × 120, 3 × 300s) and ECG-gated images with 16 bins. The ^15^O-water gated images were reconstructed to represent the blood pool using a standardized and fixed time window of 0–50 s. Since no previous studies have assessed blood pool ^15^O-water gating using more than 8 bins, an additional 8-bin reconstruction was performed for comparison. The ^11^C-acetate gated reconstructions represented the myocardial retention 2–7 min post injection. PET images were reconstructed using time-of-flight ordered subsets expectations maximization (3 iterations, 16 subsets, 5 mm Gaussian post-filter) with point spread function recovery.

### PET image analysis

**Fig. 1:**
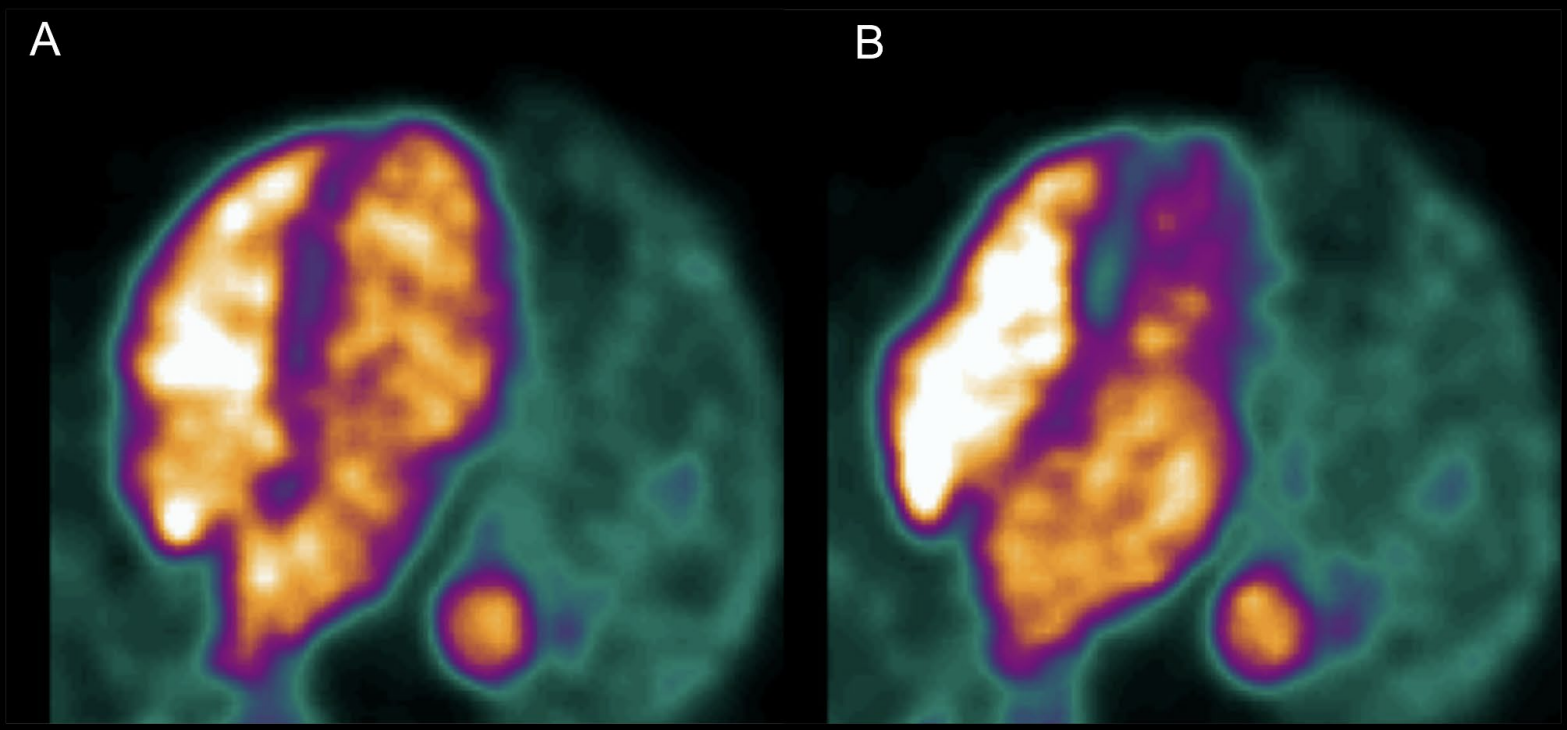
Example of blood-pool based gating in ^15^O-water PET during the end-diastolic **(A)** and end-systolic **(B)** phase

All PET images were analysed using aQuant Research (MedTrace A/S, Hørsholm, Denmark). The ^15^O-water gated analysis was based on blood-pool images and the steepest path approach, defining seed points in the image, as described previously [20, 21]. Parametric blood-volume images were derived from the single-tissue compartment model for ^15^O-water [22], allowing for automatic separation of the seeds in the left and right side of the heart. The gated analysis included only the LV. The LV was separated from the left atrium in each bin using a line placed in the valve plane, and the seeds belonging to the LV-side were used for volume calculation. Manual adjustments of the separation between left atrium and LV were performed when deemed necessary by the observer, blinded to CMR data. A representative ^15^O-water gating image is shown in Fig. 1.

The ^11^C-acetate gated analysis was fully automated and based on LV wall delineations [14], applied to all binned images. The gated analyses were used to measure LV end-diastolic volume (EDV) and end-systolic volume (ESV). Total LV stroke volume (LVSV) was defined as EDV-ESV, and LV ejection fraction (LVEF) as LVSV/EDV. An animated example of ^15^O-water and ^11^C-acetate gating in the same patient is shown Supplementary material (Figure S1).

Cluster analysis was used to extract both the arterial input function consisting of the LV and aorta, and the venous input function consisting of the right atrium, right ventricle and pulmonary trunk. Cardiac output (CO) was calculated using indicator dilution methods based on the area under the curve (AUC) of the arterial and venous input, with the formula CO = Injected tracer dose/AUC [12, 13]. Heart rate (HR) registered during the first pass was used to calculate FSV, defined as CO/HR. HR in ^15^O-water scans was derived from the ECG-gated data, as this resembled the actual first pass (0–50 s), whilst a manually recorded HR was used for the ^11^C-acetate scans. A calibration of the PET FSV was performed using linear regression of the ^11^C-acetate FSV towards CMR. The regression was subsequently applied prospectively to ^15^O-water imaging data. PET-based regurgitant volume (RegVol) was estimated by subtraction of calibrated FSV from LVSV, and subsequently, regurgitant fraction (RegF) was calculated as RegVol/LVSV.

### Cardiovascular magnetic resonance (CMR)

The CMR examinations were performed on a 3-Tesla Ingenia Philips whole body scanner (Philips Healthcare, Best, Netherlands) with an 80 mT^*^m^− 1^ gradient system. Short- and long-axis cine images were generated with a steady-state free precession pulse sequence. The basal slice was defined on long-axis images and LV geometrical volumes were manually segmented from short-axis images. End-diastolic endo- and epicardial contours were delineated, including the papillary muscle tissue in the blood pool, as according to clinical procedure. Additionally, LV volume calculations were repeated with manual exclusion of the papillary muscles from the blood pool. Aortic FSV was quantified semiautomatically with phase-contrast images acquired perpendicular to the proximal ascending aorta during free breathing. Aortic FSV was corrected for aortic backflow, to better resemble the PET-calculations. Images were analysed using commercial software (CVI42, Circle Cardiovascular Imaging, Calgary, Canada). RegVol and RegF were calculated as described in the PET image analysis section.

### Statistics

Continuous variables were tested for normal distribution using Shapiro-Wilks test and were normal distributed if not stated otherwise. Continuous variables are expressed as mean ± standard deviation (SD). Correlations between measurements were assessed with linear regression and agreement between methods with Bland-Altman analysis. Systematic and proportional bias were tested with paired t-tests and linear regression, respectively. Unpaired t-tests were used to compare PET measurements in patients and healthy volunteers. Diagnostic accuracy was evaluated with contingency tables. P-values < 0.05 were considered statistically significant. Statistical and graphical analyses were performed in JMP 17 (SAS Institute Inc., Cary, NC, USA) and GraphPad Prism version 10 (GraphPad Software, La Jolla, CA, USA).

## Results

Patient data are presented in Table 1. In total, 21 patients were included. All patients underwent ^15^O-water PET and CMR scanning, and 20 underwent ^11^C-acetate PET. One patient did not undergo ^15^O-water gated analysis due to technical issues and one other patient did not complete the cine CMR sequence for volumetric assessment. Thus, FSV was obtained with CMR and ^15^O-water in all 21 patients, and LVSV, RegVol and RegF in 20 patients. The extra CMR analysis with exclusion of papillary muscle from the blood pool was performed in 19 patients.

**Table 1:** Clinical data of patients with primary mitral valve regurgitation and healthy volunteers. Asterisk indicates statistical significance in unpaired t-test

	Patients	Healthy volunteers	*P*-value (two-tailed)
*N*	21	18	
Male sex, *n*	20	6	
Age, years	66±7	57 ± 14	0.02*
Body surface area, m^2^	2.0±0.2	1.8 ± 0.2	0.003*
Body mass index, kg/m^2^	25.4±2.5	23.5 ± 3.0	0.04*
Heart rate, beats/min	61±10	61 ± 7	0.9
Systolic blood pressure, mmHg	147±15	134 ± 15	0.01*
Diastolic blood pressure, mmHg	85±10	81 ± 11	0.2

Of the 20 patients with CMR measurements of RegVol, 16 were confirmed to have severe mitral regurgitation (> 60 mL), 3 patients were found to have moderate disease (> 30 < 60 mL) and 1 mild disease (< 30 mL).

Two healthy volunteers with imaging findings suggestive of undiagnosed cardiac disease (significant chamber dilatation, LV hypertrophy) were excluded. One healthy volunteer was excluded due to excessive motion during the PET acquisition, compromising the analysis. Ultimately, the total number of healthy volunteers included in the analysis were 18 for ^15^O-water, of which 5 also underwent ^11^C-acetate PET.

### Gated analysis and volumetric measurements

PET LV volumes correlated well with CMR for both ^15^O-water and ^11^C-acetate (EDV *r* = 0.97 and 0.95; ESV *r* = 0.75 and 0.88; LVSV *r* = 0.94 and 0.92, all *p* < 0.001) and moderately for LVEF (*r* = 0.46, *p* = 0.05 and *r* = 0.54, *p* = 0.02). PET underestimated LV volumes, while no systematic bias was present for LVEF when calculated with 16 bins. Mean ^15^O-water and ^11^C-acetate values from the gated image analyses, and results from Bland-Altman analyses of EDV, ESV, LVSV, and LVEF compared to CMR are presented in Table 2.

**Table 2:** Mean values ± standard deviation (SD) and comparison of values from volumetric CMR measurements and PET gating analysis in the mitral regurgitation patients. Bias was calculated using bland-Altman analysis and p-values were calculated with paired t-tests. EDV = end-diastolic volume, ESV = end-systolic volume, SV = total stroke volume, EF = ejection fraction

	Modality	Mean±SD	Bias ± SD (PET-CMR)	*P*-value
**EDV (mL)**	CMR	235±61		
	^15^O-water 8 bins	206±50	-31.0±21.0	< 0.001
	^15^O-water 16 bins	214±52	-22.1±17.1	< 0.001
	^11^C-acetate	203 ± 52	-36.7 ± 20.1	< 0.001
**ESV (mL)**	CMR	72±21		
	^15^O-water 8 bins	68±19	-4.6±12.9	0.13
	^15^O-water 16 bins	62±18	-10.5±14.1	0.005
	^11^C-acetate	65 ± 18	-8.2 ± 10.0	0.002
**SV (mL)**	CMR	163±44		
	^15^O-water 8 bins	138±38	-26.3±17.8	< 0.001
	^15^O-water 16 bins	153±42	-11.6±15.0	0.003
	^11^C-acetate	137 ± 38	-28.5 ± 16.6	< 0.001
**EF (%)**	CMR	70±4		
	^15^O-water 8 bins	67±6	-2.5±4.8	0.03
	^15^O-water 16 bins	71±6	1.6±5.1	0.19
	^11^C-acetate	68 ± 4	-1.7 ± 3.9	0.08

**Fig. 2:**
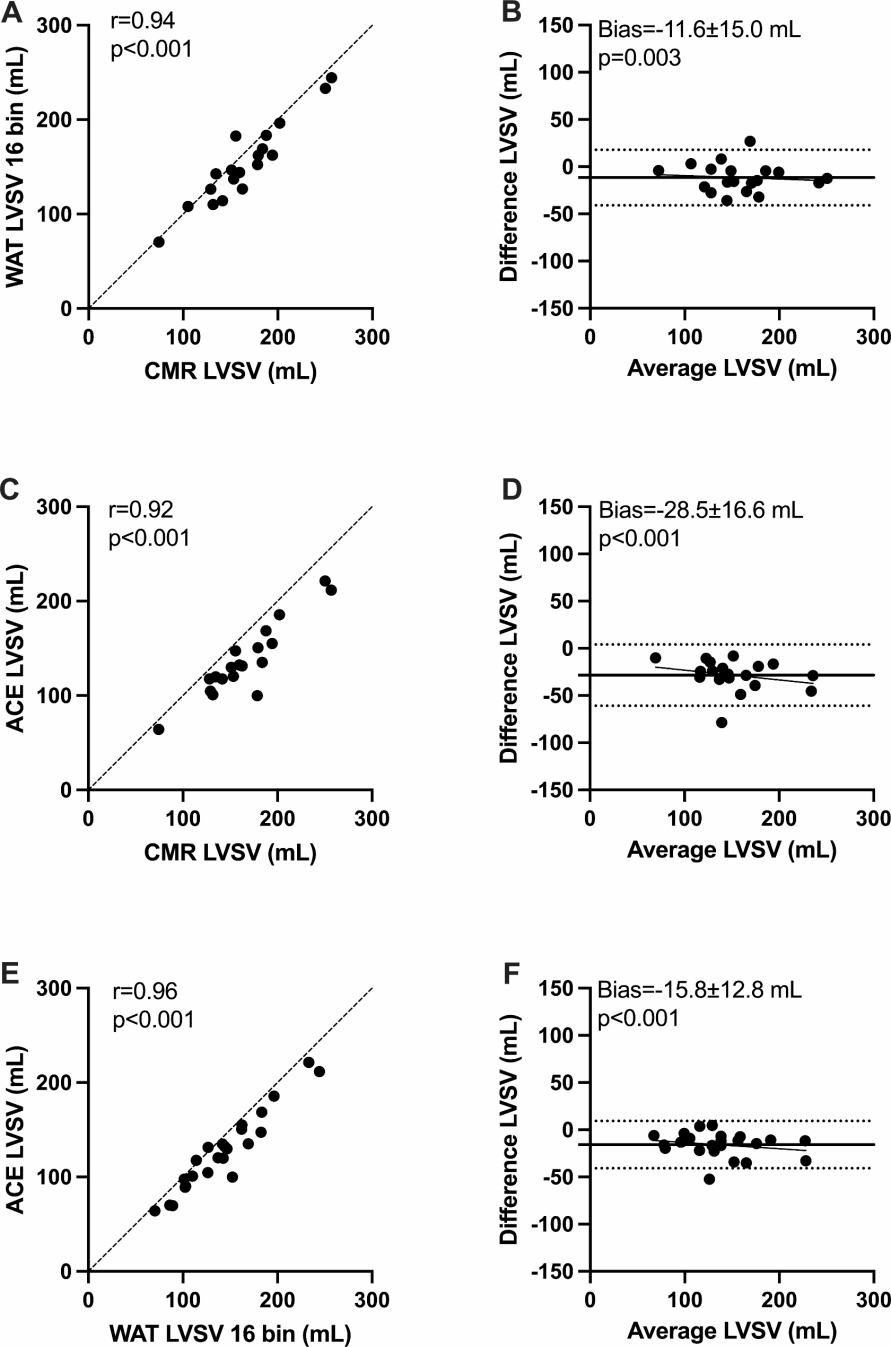
Scatter plots (left) and Bland-Altman plots (right) of left ventricular total stroke volume (LVSV) calculated with ^15^O-water (WAT), ^11^C-acetate (ACE) and cardiovascular magnetic resonance (CMR). Dashed lines are lines of identity (**A**, **C**, **E**), dotted lines limits of agreement (**B**, **D**, **F**), and solid lines represent linear regression and mean bias (**B**, **D**, **F**)

The ^15^O-water LVSV calculated with 16 bin gating showed slightly stronger correlation and closer agreement towards CMR, compared to 8 bins (*r* = 0.94 vs. 0.92, bias=-11.6 ± 15.0 mL vs. -26.3 ± 17.8 mL). Hence, the 16-bin reconstruction was used to calculate RegVol and RegF in ^15^O-water PET. A comparison between 16 bin ^15^O-water, ^11^C-acetate and CMR based LVSV is shown in Fig. 2.

### First-pass analysis with indicator dilution techniques

PET-based CO calculated from the arterial and venous input function, respectively, correlated strongly (^15^O-water *r* = 0.94 and ^11^C-acetate *r* = 0.97, *p* < 0.001) (Figure S2), and a similar result was found for FSV (^15^O-water: *r* = 0.94 and ^11^C-acetate *r* = 0.97, *p* < 0.001). For each tracer, no systematic or proportional bias was found between arterial and venous input function.

Uncalibrated PET-based FSV was significantly higher than CMR, when calculated from both arterial and venous clusters (^15^O-water arterial bias = 35.7 ± 9.7 mL, venous bias = 36.7 ± 9.4 mL and ^11^C-acetate arterial bias = 37.7 ± 12.6 mL, venous bias 35.7 ± 12.6 mL, *p* < 0.001). The ^11^C-acetate venous input function was used to calibrate all PET FSV values. Linear regression with uncalibrated FSV versus CMR resulted in an intercept of 9 and a slope of 0.59, which was applied as a calibration factor for the arterial and venous cluster in both tracers. Linear regression analyses for uncalibrated and calibrated FSV values are shown in Supplementary material (Figure S3). The calibrated FSV values from the arterial input for ^15^O-water and ^11^C-acetate were used to calculate PET RegVol and RegF.

**Fig. 3:**
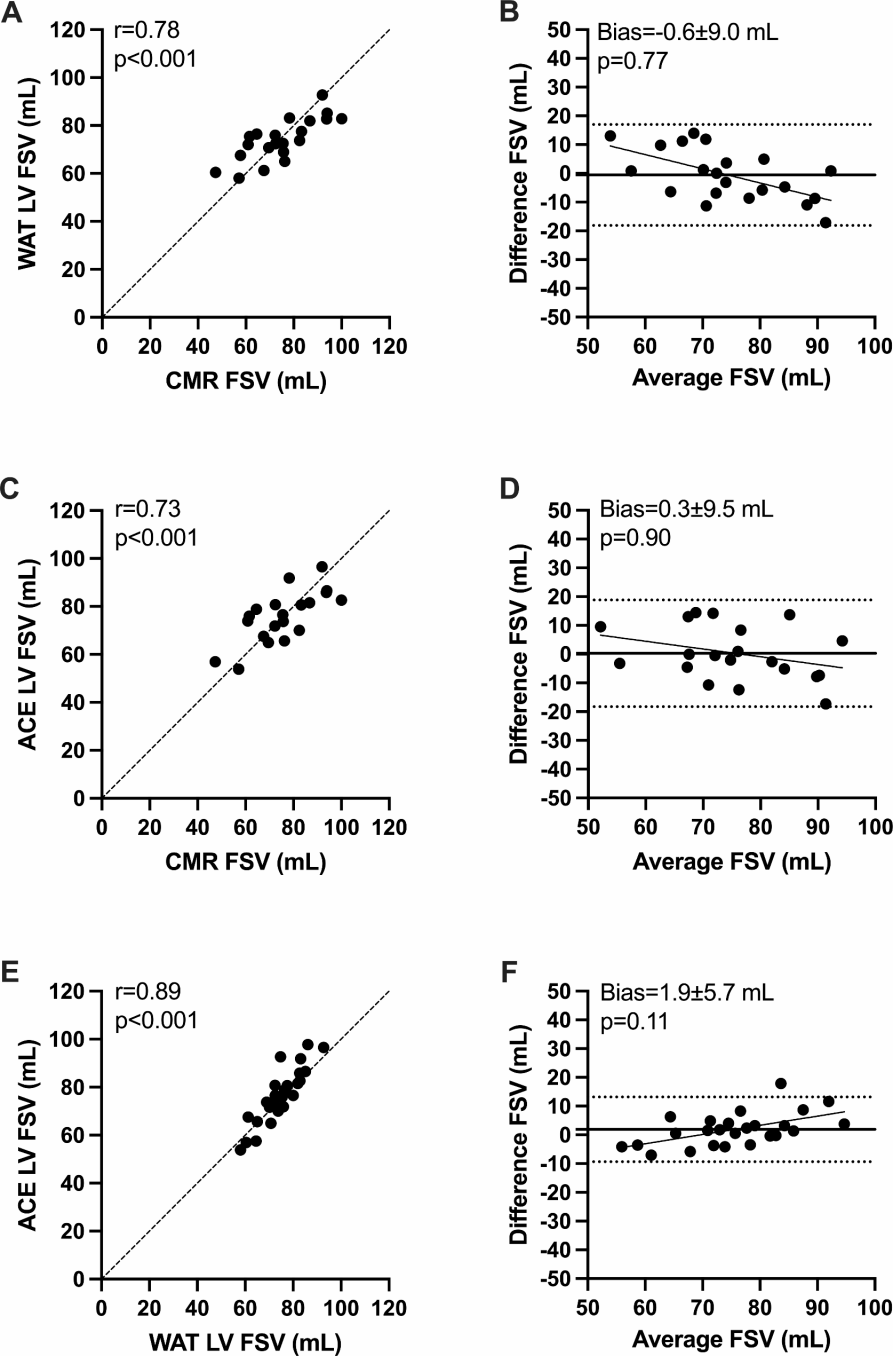
Scatter plots and Bland-Altman plots of forward stroke volume (FSV) calculated with ^15^O-water (WAT), ^11^C-acetate (ACE) and cardiovascular magnetic resonance (CMR). Dashed lines are lines of identity (**A**, **C**, **E**), dotted lines are limits of agreement (**B**, **D**, **F**), and solid lines represent linear regression and mean bias (**B**, **D**, **F**). A proportional bias between ^15^O-water versus CMR **(B)** and ^15^O-water versus ^11^C-acetate **(F)** is present

Calibrated FSV in ^15^O-water and ^11^C-acetate correlated strongly with CMR. No systematic bias was found, but a small significant proportional bias for ^15^O-water versus both CMR and ^11^C-acetate (Fig. 3).

### Regurgitant volume and fraction

**Fig. 4:**
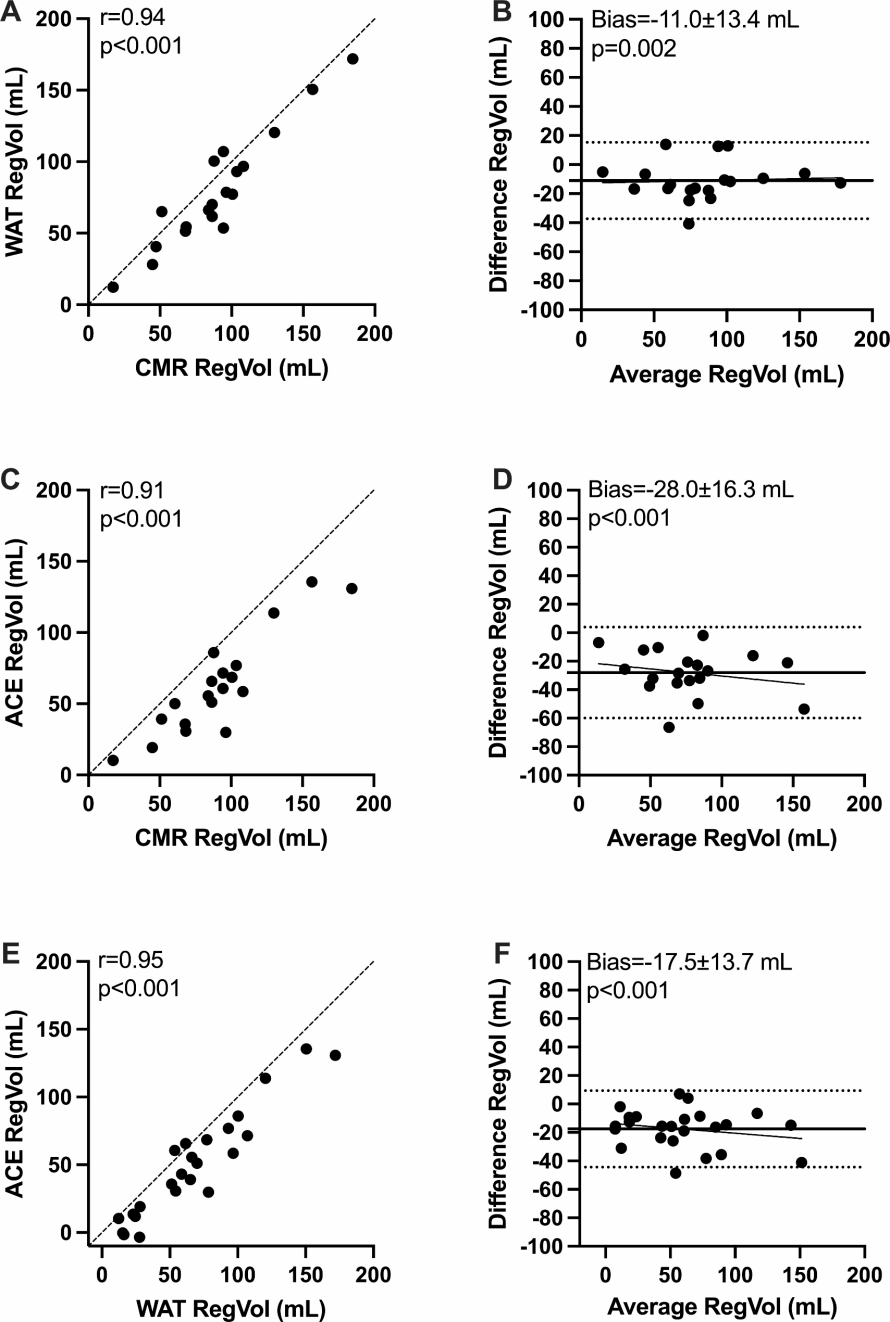
Scatter plots and Bland-Altman plots of regurgitant volume (RegVol) calculated with ^15^O-water, ^11^C-acetate and cardiovascular magnetic resonance (CMR). Dashed lines are lines of identity (**A**, **C**, **E**), dotted lines are limits of agreement (**B**, **D**, **F**), and solid lines represent linear regression and mean bias (**B**, **D**, **F**)

**Fig. 5:**
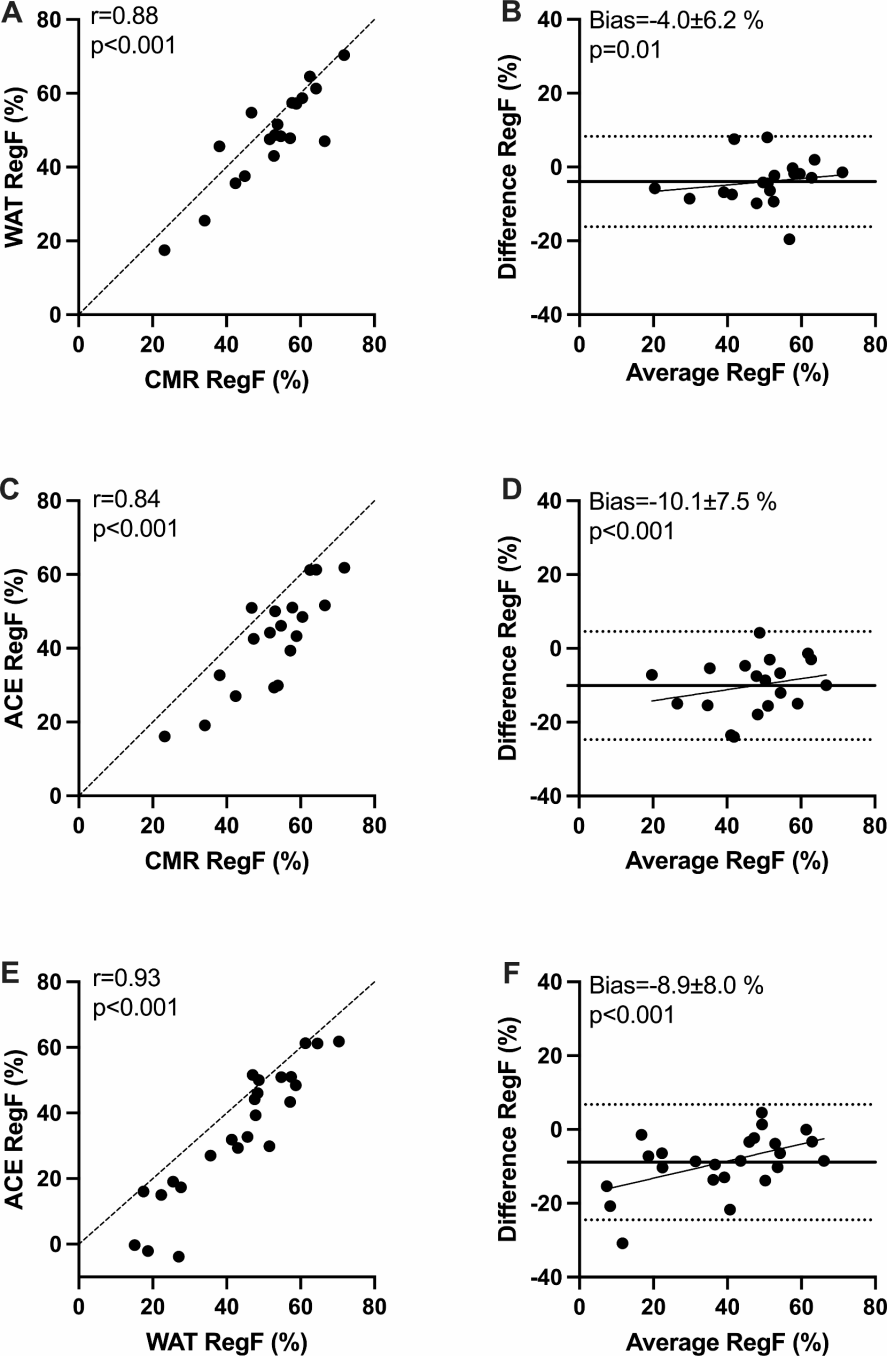
Scatter plots and Bland-Altman plots of regurgitant fraction (RegF) calculated with ^15^O-water (WAT), ^11^C-acetate (ACE) and cardiovascular magnetic resonance (CMR). Dashed lines are lines of identity (**A**, **C**, **E**), dotted lines are limits of agreement (**B**, **D**, **F**), and solid lines represent linear regression and mean bias (**B**, **D**, **F**). A proportional bias between ^11^C-acetate versus ^15^O-water **(F)** is present

Strong correlations were found for RegVol and RegF based on ^15^O-water and ^11^C-acetate PET versus CMR. For both PET tracers, RegVol and RegF were underestimated. There was a closer agreement between ^15^O-water and CMR versus ^11^C-acetate and CMR, and a significant systematic bias was present between the tracers (Figs. 4 and 5). There was a proportional bias when comparing ^15^O-water and ^11^C-acetate RegF (slope = 0.23, *p* = 0.01).

Mean values and comparisons between PET-parameters in patients and healthy volunteers are presented in Table 3. Average PET RegVol and RegF for both tracers were significantly lower in healthy volunteers compared to the patients (RegVol: ^15^O-water = 21 ± 7 vs. 78 ± 39 mL, ^11^C-acetate = 4 ± 8 vs. 62 ± 34 mL and RegF ^15^O-water = 22 ± 7 vs. 48 ± 13%, ^11^C-acetate = 5 ± 10 vs. 42 ± 13%, *p* < 0.001). Contingency tables showed that moderate mitral regurgitation (RegVol > 30 mL) was detected with an accuracy of 95% (*n* = 38, *p* < 0.001) for ^15^O-water and 92% (*n* = 25, *p* < 0.001) for ^11^C-acetate.

**Table 3:** Results from PET-measurements comparing mitral regurgitation patients and healthy volunteers: mean values ± standard deviation (SD) and two-tailed p-values from t-test between patients and healthy volunteers. MR = mitral regurgitation, HV = healthy volunteer, LV = left ventricular

	^15^O-water			^11^C-acetate		
	MR (*n* = 21)	HV (*n* = 18)	*P*-value (two-tailed)	MR (*n* = 20)	HV (*n* = 5)	*P*-value (two-tailed)
Regurgitant volume (mL)	78 ± 39	21 ± 7	< 0.001	62 ± 34	4 ± 8	< 0.001
Regurgitant fraction (%)	48 ± 13	22 ± 7	< 0.001	42 ± 13	5 ± 10	< 0.001
Forward stroke volume index (mL/m^2^)	37 ± 3	40 ± 5	0.02	37 ± 4	42 ± 5	0.1
LV total stroke volume index (LVSV, mL/m^2^)	75 ± 20	51 ± 5	< 0.001	67 ± 19	44 ± 2	< 0.001

When the LV papillary muscle volume was excluded from the CMR LVSV measurements, no significant bias was found between ^15^O-water PET and CMR for RegVol and RegF (RegVol bias=-2.7 ± 13.9 mL, *p* = 0.4 and RegF bias=-0.9 ± 5.4%, *p* = 0.5). However, a significant bias remained for ^11^C-acetate PET (RegVol bias=-21.0 ± 17.2 mL, *p* < 0.001 and RegF bias=-7.6 ± 7.5%, *p* = 0.001).

Both 8 and 16 bin based ^15^O-water LVSV correlated strongly with CMR for RegVol and RegF calculation. Using 16 bins lead to stronger correlations and higher precision versus 8 bins (RegVol: *r* = 0.94 vs. 0.90, bias=-11.0 ± 13.4 mL vs. -25.8 ± 17.1 mL and RegF: *r* = 0.88 vs. 0.82, bias=-4.0 ± 6.2% vs. 9.5 ± 8.2%).

## Discussion

Several studies evaluated PET-based LV volumes and cardiac function with ECG gating and indicator dilution techniques, but, to the best of our knowledge, this is the first study combining the two methods for quantitation of mitral regurgitation severity. The different functional assessments required for regurgitation calculation were possible to perform with a high degree of automation on data from a single PET acquisition, using either a freely diffusible (^15^O-water) or a metabolically trapped (^11^C-acetate) PET tracer with similar results. PET measurements of regurgitation magnitude correlated strongly with CMR, and using PET-based RegVol it was possible to discriminate the controls from patients with high accuracy. These results indicate that PET is able to detect and roughly quantify mitral regurgitation.

All gated PET reconstructions used in the current study can be performed automatically on the PET console, if the time window for ECG gating is pre-defined. For ^11^C-acetate, this is straightforward, as the myocardial uptake is used to delineate the cavity and calculate volumes. We chose ^11^C-acetate data between 2 and 7 min, as has been described previously [14]. During this time window, the myocardial uptake is expected to be clearly visualized, but a number of different time settings would likely be applicable. ^15^O-water blood pool gating is more sensitive, since for adequate contrast between myocardial tissue and cavity, the acquisition window has to be short enough for the bolus to remain intact during first pass, which limits the amount of counts available for calculation. The acquisition window might need adjustments for patients with unusually low or high CO [18]. A window of 0–50 s worked for the analysis of all the subjects included in the current study, and CO did not differ between patients and healthy volunteers. If the scans are acquired in list-mode, as in this study, it would be possible to reconstruct the gated series using a different acquisition interval retrospectively.

PET-based LV volumes correlated strongly with CMR, while the correlation for LVEF was only moderate. This was most likely due to the narrow range of LVEF values, and in good agreement with previously reported measurements using ECG gated PET [9]. The accuracy of gating-based volume measurements is dependent on temporal resolution and no published data have been presented where more than 8 gating bins were used for ^15^O-water PET, likely due to the use of data from older, non-digital PET scanners with relatively low sensitivity and resolution. Increasing the number of bins to 16 improved the agreement of PET-based RegVol and RegF towards CMR. A slightly higher change was seen in ESV as compared with EDV when increasing the number of gating bins. This was expected since the end-systolic phase is shorter, and the ESV is more likely overestimated when using a low temporal resolution.

PET systematically underestimated LV volumes in comparison to CMR for both tracers. However, when performing an additional CMR analysis with removal of papillary muscle volume from EDV, the bias was no longer present between ^15^O-water and CMR. Also, the underestimation for RegVol and RegF was eliminated. In hindsight, this observation suggests that CMR-based evaluation of LV regurgitation using standard circular chamber delineations overestimates RegVol. The bias for LVSV, RegVol and RegF remained between ^11^C-acetate and CMR, primarily explained by the larger underestimation of EDV in ^11^C-acetate. This underestimation for ^11^C-acetate EDV was similar to prior results [14].

The ^15^O-water and ^11^C-acetate arterial and venous based CO correlated strongly, with no bias between tracers or clusters. This was in line with the high accuracy presented in previous studies assessing CO from PET data with older scanner types [12, 13], and suggests that the method is robust and reproducible. With a recorded HR, the FSV is thereafter easily derived from the CO. Using ECG during the scan automated this process for ^15^O-water PET, since the HR registration corresponded to the first pass during which CO was measured. For ^11^C-acetate PET, the ECG-derived HR corresponded to 2–7 min into the scan, and thus it is possible that the HR recorded differs from the first pass. This was the case in 2 subjects, where the gating-based and manual HR differed 10 and 13 heart beats. Therefore, the manual recording was used for calculation of ^11^C-acetate FSV, even though there were no significant mean differences between the two methods on the group level.

As in all cases of indicator dilution techniques, correct PET-based CO calculation requires the knowledge of exact amount of injected radioactivity. Since ^15^O-water has a half-life of 2.03 min, precise measurements are challenging. The scans conducted in the current study utilized a standardized semi-automatic tracer injection. Radioactivity was measured in a syringe in a dose calibrator 2 min before injection, aiming for 830 MBq to account for isotope half-life and residual activity in the syringe and tubing, and resulting in an injected dose of approximately 400 MBq. It is difficult to obtain the precise amount for every injection and the residue depends somewhat on the concentration of the tracer produced, and the manual handling of the syringe. Using more automated boluses would likely ensure an even more robust CO calculation for ^15^O-water. With ^11^C-acetate, the residual activity in the syringe and tubing could be accounted for by direct measurements after injection. However, the actual amount of radioactivity reaching the main circulation during the first pass is still somewhat uncertain as PET images frequently show remaining activity at the injection site and brachial veins, even when flushing with 30 mL saline. This may contribute to the overestimation of FSV as shown in Figure S3.

In line with the findings in this study, earlier experiments have shown PET-based FSV utilizing 5 s frames during first pass to be overestimated in comparison to CMR [13]. The overestimation was scanner dependent, but no evaluation has previously been performed for the type of scanner used in this study. Therefore, we performed the scanner specific calibration of the FSV towards CMR using linear regression. It is a methodological limitation that cardiac output and FSV calculations currently require scanner-dependent corrections. The calibration should preferably be based on invasive thermodilution or local CMR devices and analysis tools.

In order to utilize either ^15^O-water or ^11^C-acetate, an on-site cyclotron is required, which is a barrier for widespread clinical implementation. However, as shown here with ^15^O-water PET, a technique based on first-pass analysis might allow most PET tracers to be used, provided that dynamic scanning is acquired, reliable estimates of injected dose are available, and the radiopharmaceutical is delivered as a fast and standardized bolus. Our results indicate that PET could be used for simultaneous evaluation of primary or secondary mitral regurgitation, LV dilatation and PET-specific parameters such as metabolism or ischemia. This might lead to a more comprehensive diagnosis from cardiac PET scans, while possibly speeding up the patient management. Considering the increasing burden of valvular diseases, allowing for calculation of mitral regurgitation using PET could be valuable. PET-based measurements of regurgitation could be particularly useful to discover secondary mitral regurgitation in perfusion imaging on rare occasions when echocardiography has not been performed prior to the PET-examination.

### Study limitations

Some limitations of the present study should be noted. The accuracy of quantifying low levels of RegVol and RegF was not assessed as the healthy volunteers were not examined with CMR, and few patients with mild and moderate mitral regurgitation were included. Only five of the healthy volunteers performed echocardiography, and thus for 13 of the controls included it is unknown whether any of the volunteers had symptom-free, undiagnosed valve disease that could have affected the results. The ^15^O-water based mean RegVol and RegF was relatively high and likely overestimated in the healthy volunteers (21 mL and 22% respectively). One methodological explanation is that identical settings were used in the gating analysis for patients and controls, although LV volumes are expected to be significantly higher in patients. A further explanation is that the FSV calibration was only performed on data from patients, as only they underwent both CMR and PET. These findings indicate that further development of the methods is needed, incorporating larger cohorts with variation in cardiac function and size. However, the PET measurements were able to separate with confidence patients with substantial mitral regurgitation from normal controls.

An inherent limitation of the PET method is the current inability to distinguish between aortic and mitral regurgitation, since only the total amount of blood not moving forward in the system is taken into account when calculating RegVol and RegF. This suggests that elevated levels of left ventricular regurgitation found with PET should be complemented with further cardiac imaging.

## Conclusions

Left ventricular regurgitation can be quantified from a cardiac PET examination. Regurgitant volumes and fractions calculated using two radiopharmaceuticals with different approaches to geometric assessments were reproducible and correlated strongly with gold standard CMR. Using PET-based regurgitant volume, it was possible to discriminate healthy controls from patients with high accuracy.

## Electronic supplementary material

Below is the link to the electronic supplementary material.

Supplementary Material 1: Fig. 1 – Example of gated PET images from the same patient. Left panel shows a^15^O-water gated scan with activity in the blood pool during the first 50 s of the scan. The right panel shows gating of corresponding^11^C-acetate retention images. Fig. 2 – Scatter plots and Bland-Altman plots comparing cardiac output (CO) calculated from left (LV) and right (RV) ventricular cavity input with ^15^O-water (WAT) (A, B) and ^11^C-acetate (ACE) (C, D). Dashed lines are lines of identity (A, C), dotted lines are limits of agreement (B, D) and solid lines represent linear regression and mean bias (B, D). Fig. 3 – Scatter plots of forward stroke volume (FSV) calculated with^15^O-water, ^11^C-acetate and cardiovascular magnetic resonance (CMR). Comparison between uncalibrated and calibrated PET values. Dashed lines are lines of identity.

## Data Availability

The data to support the findings in the study are available from the corresponding author upon reasonable request.
