# Supplementary material for: Quantitation of mitral regurgitation using positron emission tomography
Source: EJNMMI Res. 2024 Sep 18;14:85. doi: 10.1186/s13550-024-01150-1 (PMC11411051; doi:10.1186/s13550-024-01150-1)
Supplement: Supplementary file 1 — Supplementary Material 1: Fig. 1 – Example of gated PET images from the same patient. Left panel shows a15O-water gated scan with activity in the blood pool during the first 50 s of the scan. The right panel shows gating of corresponding11C-acetate retention images. Fig. 2 – Scatter plots and Bland-Altman plots comparing cardiac output (CO) calculated from left (LV) and right (RV) ventricular cavity input with 15O-water (WAT) (A, B) and 11C-acetate (ACE) (C, D). Dashed lines are lines of identity (A, C), dotted lines are limits of agreement (B, D) and solid lines represent linear regression and mean bias (B, D). Fig. 3 – Scatter plots of forward stroke volume (FSV) calculated with15O-water, 11C-acetate and cardiovascular magnetic resonance (CMR). Comparison between uncalibrated and calibrated PET values. Dashed lines are lines of identity. [file 13550_2024_1150_MOESM1_ESM.docx]

# Supplementary material


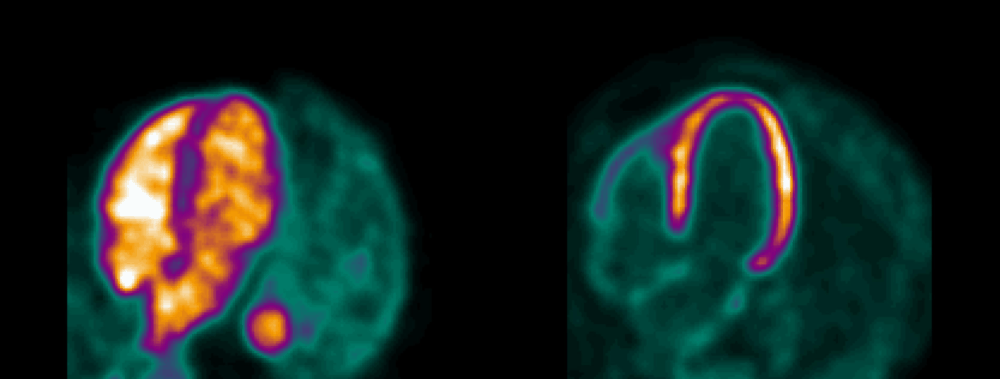


Supplementary figure 1 – Example of gated PET images from the same patient. Left panel shows a ^15^O-water gated scan with activity in the blood pool during the first 50 s of the scan. The right panel shows gating of corresponding ^11^C-acetate retention images


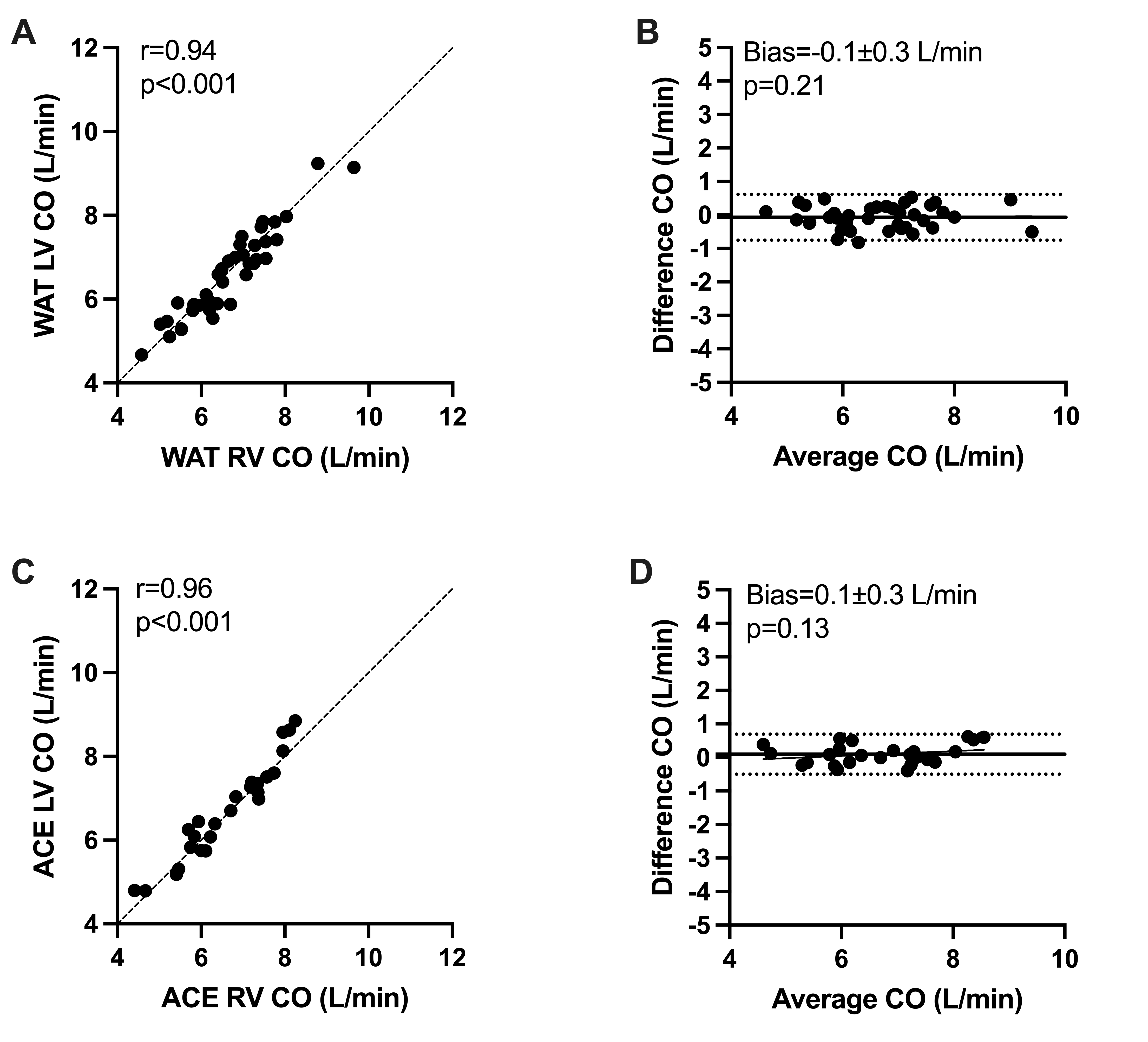


Supplementary figure 2 – Scatter plots and Bland-Altman plots comparing cardiac output (CO) calculated from left (LV) and right (RV) ventricular cavity input with ^15^O-water (WAT) (A, B) and ^11^C-acetate (ACE) (C, D). Dashed lines are lines of identity (A, C), dotted lines are limits of agreement (B, D), and solid lines represent linear regression and mean bias (B, D)


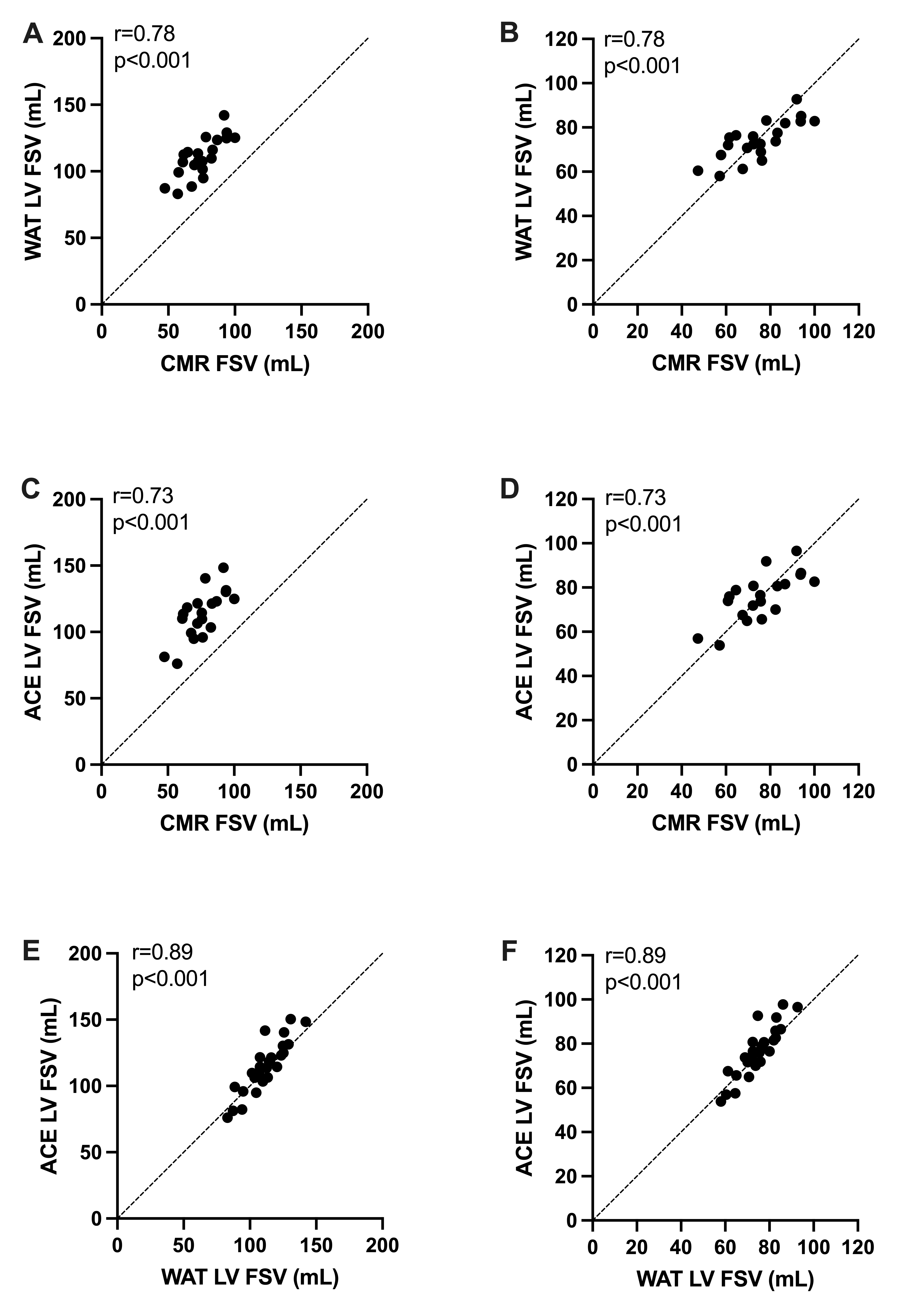


Supplementary figure 3 – Scatter plots of forward stroke volume (FSV) calculated with ^15^O-water, ^11^C-acetate and cardiovascular magnetic resonance (CMR). Comparison between uncalibrated and calibrated PET values. Dashed lines are lines of identity
